# Evaluating the Antagonistic Potential of Actinomycete Strains Isolated From Sudan’s Soils Against *Phytophthora infestans*

**DOI:** 10.3389/fmicb.2022.827824

**Published:** 2022-06-29

**Authors:** Ola Abdelrahman, Sakina Yagi, Marmar El Siddig, Adil El Hussein, Fanny Germanier, Mout De Vrieze, Floriane L’Haridon, Laure Weisskopf

**Affiliations:** ^1^Department of Biology, University of Fribourg, Fribourg, Switzerland; ^2^Department of Botany, University of Khartoum, Khartoum, Sudan

**Keywords:** *Streptomyces*, *Phytophthora infestans*, Sudan’s soils, Actinomycetes, potato, late blight

## Abstract

Soil microorganisms play crucial roles in soil fertility, e.g., through decomposing organic matter, cycling nutrients or through beneficial interactions with plants. Actinomycetes are a major component of soil inhabitants; they are prolific producers of specialized metabolites, among which many antibiotics. Here we report the isolation and characterization of 175 Actinomycetes from rhizosphere and bulk soil samples collected in 18 locations in Sudan. We evaluated the strains’ metabolic potential for plant protection by testing their ability to inhibit the mycelial growth of the oomycete *Phytophthora infestans*, which is one of the most devastating plant pathogens worldwide. Most strains significantly reduced the oomycete’s growth in direct confrontational *in vitro* assays. A significant proportion of the tested strains (15%) were able to inhibit *P. infestans* to more than 80%, 23% to 50%–80%, while the remaining 62% had inhibition percentages lesser than 50%. Different morphologies of *P. infestans* mycelial growth and sporangia formation were observed upon co-inoculation with some of the Actinomycetes isolates, such as the production of fewer, thinner hyphae without sporangia leading to a faint growth morphology, or on the contrary, of clusters of thick-walled hyphae leading to a bushy, or “frozen” morphology. These morphologies were caused by strains differing in activity levels but phylogenetically closely related with each other. To evaluate whether the isolated Actinomycetes could also inhibit the pathogen’s growth *in planta,* the most active strains were tested for their ability to restrict disease progress in leaf disc and full plant assays. Five of the active strains showed highly significant protection of potato leaves against the pathogen in leaf disc assays, as well as substantial reduction of disease progress in full plants assays. Using cell-free filtrates instead of the bacterial spores also led to full protection against disease on leaf discs, which highlights the strong crop protective potential of the secreted metabolites that could be applied as leaf spray. This study demonstrates the strong anti-oomycete activity of soil- and rhizosphere-borne Actinomycetes and highlights their significant potential for the development of sustainable solutions based on either cell suspensions or cell-free filtrates to safeguard potatoes from their most damaging pathogen.

## Introduction

The Gram positive Actinomycetes are filamentous bacteria belonging to the phylum Actinobacteria, which represents one of the most diverse groups of microorganisms in nature (van Bergeijk et al., 2020). Actinomycetes are known to be very prolific producers of specialized metabolites and they have been mainly exploited, so-far, because of their capability to produce antibiotics, as well as many anticancer, anthelmintic, and antifungal compounds. Hence, they are considered to have a major importance for biotechnology, medicine, and agriculture (Barka et al., 2016). Although few examples of plant pathogenic Actinomycetes exist, such as *Streptomyces scabies* causing common scab of potato (Keinath and Loria, 1989), many more Actinomycetes have been reported to inhibit the development of a broad range of phytopathogenic fungi (Berg et al., 2001). Among these, different species of *Streptomyces* and *Nocardiopsis* showed activity *in vitro* and *in planta* against fungal and bacterial pathogens of tomato and carrot (Djebaili et al., 2021), while *Streptomyces coelicolor* was reported to be very potent at controlling onion bacterial rot and at reducing the disease incidence throughout onions’ storage time (Abdallah et al., 2013). Furthermore, Chen et al. (2016) showed that both cells and culture filtrate of *Streptomyces plicatus* inhibited mycelial growth and zoospore germination of the pepper pathogen *Phytophthora capsici*. As a consequence of their remarkable metabolic activity, some *Streptomyces* strains are already included in commercial preparations marketed for the biological control of fungal and bacterial diseases. These preparations contain either cell suspensions, usually for soil treatments (e.g., *S. lydicus* WYEC108, the active ingredient of Actinovate, and *S. griseoviridis* K61, the active ingredient of Mycostop), or purified metabolites to be used as foliar sprays (e.g., polyoxin D, streptomycin, and kasugamycin; Rey and Dumas, 2017).

Among the many organisms endangering crop health worldwide, the oomycete *P. infestans* causing late blight in potato is a particularly devastating one, with global economic losses estimated at about 9 billion euros per year (Haverkort et al., 2009). As many other diseases, potato late blight is controlled by multiple applications of synthetic pesticides in conventional agricultural management, and by copper-based products in organic potato production, which both have negative side effects on environmental and human health (De Vrieze et al., 2018). Therefore, sustainable approaches for late blight management are needed in order to minimize crop losses as well as to decrease the environmental problems posed by fungicide applications (Majeed et al., 2017). In view of their above-mentioned prolific production of bioactive metabolites, Actinomycetes appear as promising candidates to contribute to sustainable solutions for late blight control. Indeed, earlier studies have reported inhibition of *P. infestans* mycelial growth by different *Streptomyces* strains or their metabolites (Sinha et al., 2014; Bermejo et al., 2020; Feng et al., 2021). However, *in vitro* inhibition activities on *P. infestans* are no warranty for the ability of the strains to restrict disease progress on plant material such as leaf discs or full plants, which in turn do not warrant efficacy in the field (Guyer et al., 2015; Hashemi et al., 2021). To the best of our knowledge, only two studies reported protective potential of Actinomycetes against *P. infestans* in full plant assays, the first on tomato and chili (Loliam et al., 2012), and the second on potato tubers (Feng et al., 2022). This latter study demonstrated substantial reduction of *P. infestans* growth in both *in vitro* and tuber inoculation assays and a small but significant tuber disease index reduction also under field conditions with one of the treatments consisting of a mixture of eight different strains. This very recent study brings promising first evidence of potato tuber protection against late blight disease by Actinomycete strains, although their ability to limit disease progress on leaves remains to be investigated. Therefore, this study aimed at evaluating the antagonistic activity of ca. 170 Actinomycetes isolated from soil and rhizosphere samples collected in different locations in Sudan, against *P. infestans* using *in vitro* assays as a first screening and *in planta* assays with selected candidate strains and their cell-free filtrates as a second step on the long road toward the development of sustainable control of potato late blight.

## Materials and Methods

### Collection and Preparation of Soil Samples

Soil samples were collected from various locations in Sudan including Port Sudan, Erkowit (Red sea State), Khartoum, Khartoum North, Omdurman (Khartoum State), Er Roseires Dam (Sennar State), Wad Albaseer, Al Bageir (Gezira State), and different areas in Dongola (Northern State). Soils were sampled from diverse habitats (plant rhizosphere, agricultural soil, and river sediments). Soil samples were mixed with CaCO_3_ (1:10 w/w; Sigma-Aldrich) and dried at 45°C for 1 h to favor Actinomycetes over fungi and other bacteria (El-Nakeeb and Lechevalier, 1963).

### Isolation of Actinomycetes

Isolation of Actinomycetes was performed by serial dilution and spread plate technique (Kumar et al., 2012). One gram of each soil sample was suspended in 9 ml of sterile distilled water and allowed to settle for 30 min (El-Nakeeb and Lechevalier, 1963). The dilution was carried out up to 10^−6^ by transferring 1 ml from the top of the soil suspension into 9 ml sterile distilled water in a sterile tube. Aliquots (0.5 ml) of the dilutions 10^−4^, 10^−5^, 10^−6^ were inoculated on Starch Casein Nitrate Agar (SCNA). SCNA medium was prepared by dissolving, in 1 L distilled water, 18 g agar (Carl Roth GmbH), 10 g starch (Sigma), 2 g NaCl (Acros organics), 2 g KNO_3_ (Carl Roth GmbH), 2 g K_2_HPO_4_ (Siegfried Zofingen), 0.05 g MgSO_4_.7H_2_O (Carl Roth GmbH), 0.01 g FeSO_4_.7H_2_O (Fluka), 0.02 g CaCO_3_ (Sigma-Aldrich), and 0.3 g casein (AppliChem). The medium was then autoclaved for 15 min at 121°C and 15 psi. To minimize the fungal growth, 10 ml/l of fungistatin (Delta Pharma) were added to the sterilized medium under aseptic conditions. The plates were gently rotated and were then incubated at 30°C for 10 days. Based on the colony morphology, the Actinomycetes cultures were selected and repeatedly sub-cultured on SCNA for purification. A Gram stain was performed for the pure cultures and each culture of Gram-positive filamentous bacteria was preserved in a slanted way in a glass vial containing SCNA medium and stored at 4°C until further studies. In addition, a glycerol stock of each isolate was prepared by inoculating the mycelium in 80% SCNB and 20% glycerol (Sigma) and maintained at −20°C for long-term preservation of the isolates.

### Morphological and Physiological Characterization of Actinomycetes

Morphological and physiological features of the isolated Actinomycetes were determined following the International Streptomyces Project (ISP) methods described by (Shirling and Gottlieb, 1966) and briefly detailed below.

#### Aerial Mass Color and Reverse Side Pigments (Color of Substrate Mycelium)

The Actinomycetes isolates were cultured in yeast extract malt extract agar medium (ISP medium No. 2), prepared by dissolving 10 g malt extract (LobaChemie), 4 g yeast extract (LobaChemie), 4 g dextrose (Carl Roth GmbH), and 20 g agar (Carl Roth GmbH) in 1 L distilled water. Aerial mycelium color was recorded after the strains had reached abundant sporulation. In cases where the aerial mycelium color fell between two colors series, both colors were recorded. The color of the substrate mycelium was determined by observing the reverse (bottom) side of mycelium growth on the ISP 2 medium.

#### Melanoid and Diffusible Soluble Pigments

The isolates were cultured in Tyrosine Agar medium (ISP 7) and incubated for 5 days at 30°C. ISP 7 was prepared by dissolving 15 ml glycerol (Sigma), 1 g L-asparagine (Fluka), 0.5 g L-tyrosine (Sigma), 0.5 g K_2_HPO_4_ (Siegfried Zofingen), 0.5 g MgSO_4_.7H_2_O (Carl Roth GmbH), 0.5 g NaCl (Acros organics), 0.01 g FeSO_4_.7H_2_O (Fluka), 20 g agar (Carl Roth GmbH), and 1 ml of trace salt solution in 1 L of distilled water. The trace salt solution was prepared by dissolving 0.1 g FeSO_4_.7H_2_O (Fluka), 0.1 g MnCl_2_.4H_2_O, 0.01 g ZnSO_4_.7H_2_O (Merck) in 100 ml distilled water. Each isolate producing a brown diffusible pigment into the medium was recorded as a melanoid pigment producer. On the other hand, the ability to produce soluble pigments other than melanoids was noted for each isolate and the pigment color was determined.

#### Spore Chain Morphology

The spore-bearing hyphae characteristics were determined by direct microscopic examination of the spore-bearing hyphae of well sporulating cultures. This was done by inserting a sterile cover slip, at an angle of 45°, into ISP two plates, after which the Actinomycetes isolates were inoculated to the contact line of the immersed cover slip and the plates were incubated at 28°C for 21 days. The cover slip was then removed and placed on a glass slide and examined under a binocular microscope. The spore-bearing hyphae of each isolate was noted as straight or Rectus (R), flexible or Flexibilis (F), Retinaculum-Apertum (RA), Monoverticillus (MV), and spiral or Spira (S).

#### Carbon Source Utilization

Actinomycetes isolates were tested for their abilities to utilize nine sugars as sole carbon source using ISP 9 medium. This medium consists of mineral salt agar as a basal medium. The basal mineral salt agar was prepared by dissolving 2.64 g (NH_4_)_2_SO_4_ (Carl Roth GmbH), 2.38  g KH_2_PO_4_ (Carl Roth GmbH), 5.65 g K_2_HPO_4_.3H_2_O (Merck), 1  g MgSO_4_.7H_2_O (Carl Roth GmbH), 15 g agar (Carl Roth GmbH), and 1 ml Pridham and Gottlieb trace salts solution prepared by dissolving 0.64 g CuSO_4_.5H_2_O (Fluka), 0.11 g FeSO_4_.7H_2_O (Fluka), 0.79 g MnCl_2_.4H_2_O (Carl Roth GmbH) and 0.15 g ZnSO_4_.7H_2_O (Merck) in 100 ml distilled water. The medium was cooled, after autoclaving, to 60°C and each sterilized carbon source was added aseptically to the basal mineral salt agar giving a final concentration of 1%. L-arabinose (Fluka), D-xylose (Fluka), D-mannitol (Sigma-Aldrich**)**, D-fructose (Fluka), rhamnose (Carl Roth GmbH), raffinose (Merck), sucrose and glucose (Carl Roth GmbH) were filter sterilized using 0.2 μm sterile filters. Cellulose (Schleicher & Schuell) and meso-inositol (Fluka), which are not sufficiently soluble to be sterilized with this method, were sterilized by the ether sterilization method (Shirling and Gottlieb, 1966). In this method, the dry carbon source was weighted and spread as a shallow layer in a pre-sterilized Erlenmeyer flask fitted with a loose cotton plug. Sufficient acetone-free ethyl ether (C_2_H_5_)_20_ (Fluka) was then added to cover the carbohydrate. The ether was allowed to evaporate at room temperature under a ventilated fume hood for 2 days, and then sterile distilled water was aseptically added to make a 10% w/v solution of the carbon source. Plates were examined after 13 days. The growth of each isolate was compared with the growth on the two control plates: the growth on the basal medium alone, without any carbon source (negative control), and the growth on the basal medium supplemented with glucose (positive control). Results were taken as follows: Strongly positive utilization (++) when the isolate growth on the medium supplemented with the tested carbon source was equal or better than the positive control; positive utilization (+) when the growth was better than the negative control, but less than the positive control; and doubtful utilization (±) when the growth was slightly better than the negative control. Carbon source utilization was recorded as negative when the growth was not better than the negative control.

### Identification of the Isolated Strains

#### DNA Extraction

DNA was extracted from the Actinomycetes using heat shock: for each strain, mycelium was transferred to a sterile 1.5 ml tube, ground using a sterile tip, and suspended thoroughly in 1 ml distilled water. The samples were heated at 95°C in the heating block for 20 min and were then suddenly transferred into ice for 10 min. The samples were then reheated in the heating block at 95°C for 10 min and the so obtained cell lysate was stored at −20°C.

#### Amplification of the 16S rDNA Gene

Polymerase chain reaction (PCR) was performed to amplify the fragment of 16S rDNA gene, using the universal primers: 27F (5′-AGA GTT TGA TCC TGG CTC AG-3′) and 1492R (5’-CGG TTA CCT TGT TAC GAC TT-3′) purchased from Microsynth. PCR amplification was performed in 25 μl of reaction mixture which contained 5 μl of cell lysate of each isolate, 0.5 μl of each primer (25 μM), 12.5 μl of Accustart II PCR toughmix (Quantabio), 0.5 μl of track loading dye (Quantabio), and 6 μl of sterile distilled water. The thermal cycler was programmed with the following conditions: an initial denaturation step at 94°C for 3 min, then 35 cycles of three steps made up of a denaturation step at 94°C during 30 s, an annealing step at 56°C during 30 s and an extension step at 72°C during 1 min 40 s, followed by a final extension step at 72°C during 10 min.

#### Agarose Gel Electrophoresis

The PCR products were examined by standard gel electrophoresis using 1% agarose gel. The gel was prepared by dissolving 0.6 g agarose in 60 ml of 1X TAE buffer (40 M Tris-acetate, and 1 mM EDTA, pH 8.0). The gel was stained with three μl of green safe gel stain (Canvax) and was then immersed in 1X TAE buffer. Three ml of each PCR product were loaded into the wells. A one Kbp-DNA ladder was used as a molecular weight marker. One hundred volts of electric current was passed through the gel for 35 min. The gel was removed, visualized in a transillumination cabinet, and images were captured using a gel documentation system. The appearance of the target band specified for the primer set (1.5 Kbp) on the agarose gel was considered as an indication of a positive amplification product.

#### Partial Sequencing of 16S rDNA Gene and Phylogenetic Analysis

PCR products were purified using a centrifugation protocol according to E.Z.NA. Cycle Pure Kit (Omega, bio-tek. United States) and following the manufacturer’s protocol, except that sterile distilled water was used to elute the DNA instead of the provided elution buffer. Twelve μl of each purified PCR product were mixed with three μl of the forward primer (27F) and sent to Microsynth Company for sequencing (Switzerland). The partial 16S rDNA gene sequences were compared with available databases using nucleotide BLAST search in NCBI GenBank. The sequences were deposited in GenBank (ON041455-ON041629). A phylogenetic tree based on the sequences of 172 isolates was constructed using the following parameters in the Geneious software: a global alignment with free end gaps was performed, with a similarity index of 65%. A neighbor-joining tree was then constructed using the Tamura-Nei genetic distance model.

### Screening of Actinomycetes Activity Against *Phytophthora infestans*

To explore the strains’ potential to inhibit *P. infestans* mycelial growth, full plate dual assays were performed on V8 plates. This type of assays enables the exchange of both volatile and diffusible compounds between the two organisms (Bruisson et al., 2019). V8 medium was prepared by adding 100 ml V8 juice (Campbell Soup Company’s), 1 g of CaCO_3_ and 15 g of agar (Carl Roth GmbH) in 1 L distilled water (Miller, 1955). Actinomycetes spore suspensions were prepared by culturing the strains on V8 plates. After the strains had reached abundant sporulation, distilled water was added to the plate and the aerial mycelium was then scraped using a sterile loop to liberate the spores into the water. The suspended spores were poured to a sterile 50 ml tube and then mixed vigorously to break the spore chains and obtain a homogeneous suspension (Shepherd et al., 2010). This suspension was filtered using fiberglass wool and then centrifuged at 5’000 rpm for 10 min. The supernatant was removed and the spores were suspended in 1 ml of sterile distilled water. Optical density (OD) was measured at 600 nm to measure the spores’ concentration and was adjusted to one with sterile distilled water. Three drops of 10 μl of the strains’ spore suspension (OD_600_ = 1) were inoculated at the border of a V8 plate, together with a plug of 5 mm of a 2-week-old *P. infestans* plate at the center of the plate [Rec01, a polyspore isolate of mating type A2 originally isolated by H. Krebs, Agroscope and described for its distribution, virulence and pathogenicity in (De Vrieze et al., 2019)]. Control plates were inoculated only with a plug of *P. infestans*. The plates were parafilmed and incubated at 21^°^C. Pictures were taken after *P. infestans* covered the entire control plates. The area covered by the mycelium was measured using the image analysing software Image J (Schneider et al., 2012) and compared with that of the control. Growth inhibition percentage was calculated for each strain as described previously (Bruisson et al., 2019). A two-tailed Student’s t-test was performed to analyze the results statistically. Moreover, the mycelium growth of *P. infestans* of each treatment showing abnormal phenotype was observed microscopically using bright field binocular microscope (Leica).

### Screening of Siderophore Production

Siderophore production of the strains was determined using a V8-based chrome azurol S (CAS) medium. The coloration solution (blue dye) was prepared by mixing two solutions: the first solution was prepared by dissolving 60.5 mg CAS (Kodak) in 50 ml distilled water, while 72.9 mg of HDTMA (C_19_H_24_BrN; Sigma-Aldrich**)** were dissolved in 40 ml distilled water to prepare solution two. Ten ml of iron solution were added to solution two before mixing with solution one. The iron solution was prepared by dissolving 27 mg FeCl_3_.6H_2_O (Merck), and 1 ml HCl (1 M) in 99 ml distilled water (Louden et al., 2011). 450 ml of V8 medium was prepared and supplemented with 15.12 g 1,4-piperazinediethansulfonic acid (Sigma), after which the pH was adjusted to 6.8 using NaOH. V8 medium and the blue dye were autoclaved separately, cooled down to 50° C and then 50 ml of the blue dye were added to the V8 medium under continuous mixing. One drop of 10 μl of each strain’ spore suspension (OD_600_ = 1) was inoculated on a CAS-V8 plate and incubated at 28°C for 7 days. Strains showing a halo zone of yellow-orange color around the colony were considered positive for siderophore production.

### Leaf Disc Assay

A leaf disc experiment was performed for Actinomycetes strains which were able to inhibit the mycelial growth of *P. infestans* to more than 90% on full plate assays. For each treatment, 15 leaf discs were cut from the third and fourth leaves of 15 potato plants grown in the greenhouse (aged ~5 weeks, Bintje cultivar) to reduce the plant variability. The leaf discs were placed on 0.7% water agar plates and the plates were put in the fridge for 1 day prior to the infection to let the leaf discs recover from the wound. *P. infestans* zoospores were harvested using a cold shock method described earlier (De Vrieze et al., 2018). Zoospores (40,000 zoospores/ml) and Actinomycetes spores (OD_600_ = 2) were mixed in a 1:1 ratio to have a final concentration of 20’000 zoospores/ml and OD_600_ = 1 Actinomycetes spore suspension. The abaxial surface of each leaf disc was inoculated with 10 μl of the mixture (Anand et al., 2020). Two controls were used: an infected control, in which the leaf discs were infected with zoospores without adding the bacteria, and a non-infected control, in which the leaf discs where neither infected with zoospores nor inoculated with bacteria. A similar protocol was used to test the activity of cell-free filtrate (CFF) from selected strains. The CFF was prepared by inoculating 1 ml of bacterial spore suspension (OD_600_ = 1) in 100 ml V8 broth medium and incubating the strains’ liquid cultures under shaking (180 rpm) for 7 days at 28° C, followed by sterile filtration of the spent medium using 0.2 μm sterile filters. The CFF was mixed in a 1:1 mixture as described above for the bacterial spore suspension. Filtered V8 broth medium was added as a third control on this CFF activity assay. The plates were then incubated in a humid chamber (polystyrene box with wet papers on the bottom) at 21°C for 5 days, after which pictures were taken, and the area covered by the mycelium on each leaf disc was measured using ImageJ following a macroinstruction developed in (Guyer et al., 2015). A two-tailed Student’s *t*-test was performed to analyze the results statistically.

### Full Plant Assay

To examine the ability of five selected strains to control late blight infection in more natural conditions, a full plant infection assay was carried out with greenhouse-grown potato plants (aged ~5 weeks, Bintje cultivar). Each strain’s spore suspension (OD_600_ = 0.5 in sterile water) was sprayed as a foliar treatment on three replicate potato plants until full coverage of the plant was reached, corresponding to a volume of ~10–15 ml/plant. The plants were incubated for 3 days in the greenhouse, after which they were moved to an infection room (at 19°C and >80% humidity) and sprayed with a *P. infestans* zoospore solution (50’000 zoospores/ml) until full coverage of the plant was reached. Two controls were used: an infected, non-treated control (sprayed with sterile water instead of bacterial suspension), and a non-infected, non-treated control (sprayed with sterile water instead of zoospore and bacterial suspension). The percentages of infected leaves were recorded 7 days after infection. Each infected leaf was given a score ranging from 1 to 5 as follows: score 1 for an infected leaf with a lesion diameter smaller than 1 cm, score 2 for a lesion diameter larger than 1 cm, score 3 for a dead leaflet, score 4 for more than one dead leaflet and score 5 was given for each dead leaf (Supplementary Figure S1). A global infection score was then calculated for each plant by multiplying the infected leaf percentages by their scores. This experiment was replicated twice independently, and the disease progress was monitored 14 days post infection. One-way analysis of variance (ANOVA) test with Tukey’s honestly significant difference test (Tukey’s HSD) was performed to determine whether there were statistically significant differences between the treatments.

## Results

### Isolation, Characterization, and Identification of Actinomycetes

**Figure 1:**
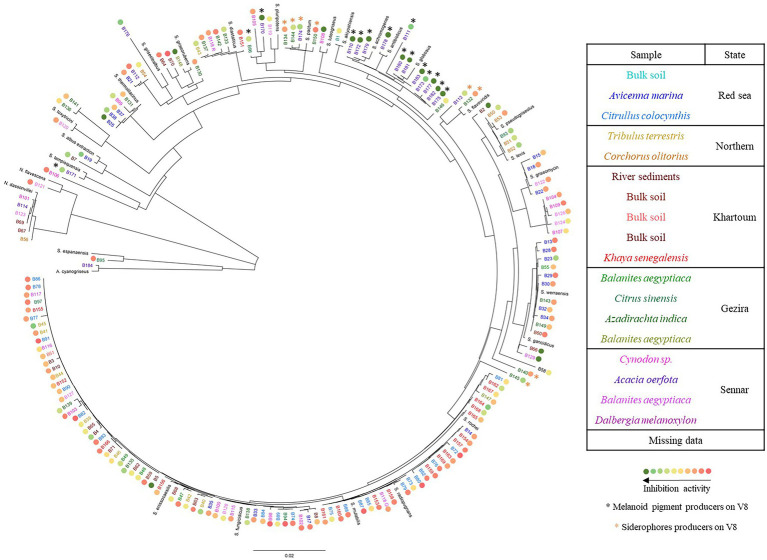
Phylogenetic tree of the isolated Actinomycetes. The tree was constructed using the Geneious software and based on ca. 1,150 bp of the 16S rDNA gene sequence. Isolates from the same soil sample are shown in the same color. The colored circles ranging from green to red indicate the strains’ inhibition activity against the mycelial growth of *Phytophthora infestans*, the black asterisks indicate the strains which produce melanoid pigments when growing on V8 (see next section) and the orange asterisks indicate the strains which produce siderophores. Reference sequences from different species within *Actinoalloteichus*, *Saccharothrix*, *Nocardiopsis* (*N*.), and *Streptomyces* (*S*.) genera corresponding to the following accession numbers were included: *Actinoalloteichus cyanogriseus* (HG917901.1), *Saccharothrix espanaensis* (AB248288.1), *Nocardiopsis dassonvillei* (MN108027.2), *N. flavescens* (KU973976.1), *S. achromogenes* (LC535408.1), *S*. *akiyoshiensis* (KU324432.1), *S. albus* (MW217110.1), *S. antibioticus* (EU841627.1) *S. diastaticus* (MN422355.1), *S. enissocaesilis* (FJ532468.1), *S. flavoviridis* (KJ685808.1), *S*. *fungicidicus* (MF120518.1), *S. gancidicus* (NR_041179.1), *S. globosus* (HM230830.1), *S. griseoloalbus* (MT669308.1), *S. griseomycini* (KX352780.1), *S. griseorubens* (KY783422.1), *S. levis* (KC848662.1), *S. luteogriseus* (LC072712.1), *S. maritimus* (MT279914.1), *S. mutabilis* (MT355865.1), *S. pactum* (EU570680.1), *S*. *pluripotens* (KX129895.1), *S. pseudogriseolus* (KT588653.1) *S. radiopugnans* (MT669290.1), *S. rochei* (MT568582.1), *S. tempisquensis* (KF954543.1), *S*. *thermolilacinus* (MN181426.1), *S. toxytricini* (KY007175.1) and *S. werraensis* (MK825539.2).

Eighteen soil samples were collected from various locations in Sudan. 175 Actinomycetes isolates were recovered, 41 were isolated from the Red Sea State, 41 from the Khartoum State, 47 from the Sennar State, 30 from the Gezira State and 15 from the Northern State (Supplementary Table S1). All the isolates were considered as Actinomycetes based on their filamentous growth (Supplementary Figure S2). The presence of Actinomycetes in all soil samples collected from different geographical regions indicated their wide distribution in different locations and habitats. The aerial mycelium color, reverse side pigment and spore chain morphology of each strain were recorded, as well as the ability to produce melanoid and other diffusible soluble pigments (Supplementary Table S2). The morphology of the spore bearing hyphae was flexibilis (flexible) for 96 isolates, retinaculum-apertum (open loops, hooks, or extended spirals of wide diameter) for 65 isolates, monoverticillus (primary verticals or whorls distributed on a long axis or branch) for seven strains, rectus (straight) for six isolates and spira (simple spiral) for one isolate. Ninety-eight strains (56% of the collection) were able to produce melanoid pigments on ISP7 medium, and they came from the 18 different soil samples. Interestingly, from the 20 strains isolated from the rhizosphere of *Acacia oerfota*, all but one (B184, which produced a violet pigment) produced melanoid pigments on ISP7 medium, while the proportion was lower in the rhizosphere of other plants. Moreover, 17 strains produced yellow (B7, B15, B57, B122, B139, B142 and B144), red (B50, B51, B52, B53, B93, B106, B130), green (B14 and B58) and yellowish red (B43) soluble pigments on ISP7 medium. Those strains were isolated from nine different soil samples. Actinomycetes isolates were tested for their abilities to use different carbohydrates as sole carbon source. More than 80% of the isolates were able to utilize arabinose (84%), raffinose (83%), mannitol (82%), and sucrose (82%). Xylose and fructose were utilized by 77% of the tested isolates, whereas cellulose and rhamnose were utilized by 67% and 57% of the isolates, respectively. Inositol was utilized by only 51% of the isolates (Supplementary Table S3). The isolated Actinomycetes strains were identified to the genus level by partial 16S rDNA sequencing. Among them, a vast majority (166) belonged to the genus *Streptomyce*s, seven to the genus *Nocardiopsis*, one was identified as *Saccharothrix espanaensis* (B95), and one as *Actinoalloteichus cyanogriseus* (B184). Among the seven *Nocardiopsis* spp., there were six strains belonging to the species *Nocardiopsis dassonvillei* (B56, B67, B69, B101, B114, B123), which is known to contain opportunistic human pathogens (Bennur et al., 2015). Each *Nocardiopsis* strain was isolated from a different soil sample, except for B121 (*N. flavescens)* and B123 (*N. dassonvillei*), which were both isolated from the rhizosphere of *Dalbergia melanoxylon* located in Sennar State (Supplementary Table S1). Among the vast majority of *Streptomyces* isolates, different subclades were found as shown in Figure 1. One subclade was exclusively composed of isolates from the rhizosphere of *Acacia oerfota* in Er Roseires. These strains clustered with *S. globosus*, *S. antibioticus,* and *S. achromogenes*. In addition, all the members of *Citrullus colocynthis* (21 members) and *Khaya senegalensis* except B151 (18 members) rhizospheres clustered together in the same subclade with *S. enissocaesilis*, *S. fungicidicus*, *S. radiopugnans*, *S. rochei*, and *S. mutabilis*. In most cases, the strains which shared the same morphology clustered together in the same subclade despite the difference in their sample origin, suggesting their widespread distribution across the sampled sites. This was observed for the red *Streptomyces* strains (B50, B51, B52, B53 and B93), which clustered with *S. levis* and *S. pseudogriseolus*, the orange *Streptomyces* strains (B25, B37, B38, B99, and B131), which clustered with *S. thermolilacinus*, the yellow *Streptomyces* strains (B15, B18, B22 and B122), which clustered with *S. griseomycini* and the white *Streptomyces* strains (B7 and B19), which clustered with *S. albus*.

### Effect of Actinomycetes on *Phytophthora infestans* Mycelial Growth

**Figure 2:**
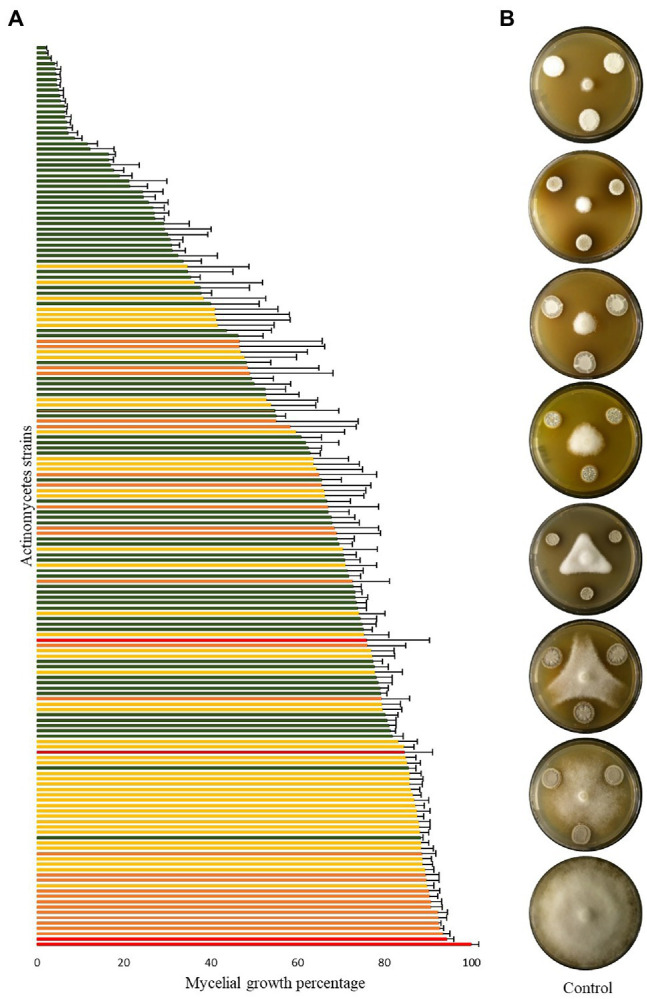
Inhibition of *Phytophthora infestans* mycelial growth by Actinomycetes strains. **(A)** Bar chart showing *P. infestans* mycelium growth when co-cultured with the different isolates, as percentage of the control growth. Bars are averages of six replicates from two independent experiments with standard error, green, yellow, and orange bars represent statistically significant differences in comparison to the control (Student’s *t*-test; *p* < 0.001, *p* < 0.01, and *p* < 0.05, respectively), while red bars represent non-significant differences to the control. The growth of the control (red bar at the bottom of the chart) was set to 100%. **(B)** Representative plates of different strains showing various *P. infestans* growth percentages compared to the control (100% growth percentage).

Most strains significantly reduced the mycelial growth of *P. infestans* in *in vitro* dual assays, although to a varying degree. As shown in Figure 2, an activity gradient was observed, with growth inhibition percentages ranging from 6% to 98%. Out of the 168 strains tested, 18 were able to inhibit *P. infestans* to more than 90%, seven to 80%–90%, 11 to 70%–80%, 13 to 60%–70%, 15 to 50%–60%, while 104 strains had inhibition percentages below 50% (Supplementary Table S4). Interestingly, a group of 15 strains clustering together with *S. pluripotens*, *S. tempisquensis*, *S. globosus, S. antibioticus* and *S. achromogenes* and all coming from the same environment (*Acacia oerfota* rhizosphere in Sennar state) displayed very high activity against *P. infestans* (Figure 1 and Supplementary Figure S3). These strains (labeled with a black asterisk in Figure 1) all produced melanoid pigments on the V8 medium used for the *P. infestans* mycelium inhibition assay, while the other strains which produced such pigments on ISP7 medium (Supplementary Table S2) did not do so when grown on V8. Eleven of these strains (B110, B170, B172, B175, B177, B178, B179, B180, B181, B182, and B183) induced more than 90% inhibition of mycelium growth, while the other four caused an inhibition of 88% (B111), 83% (B173), 73% (B171) and 62% (B96; Supplementary Figure S3). Among the many isolates belonging to the *Streptomyces* genus, the active inhibitors of *P. infestans* growth were widely distributed within the phylogenetic tree, except for the melanoid pigment producers on V8 medium mentioned above, which clustered together. Interestingly, the two most active strains, B5 and B108, which inhibited 98% of *P. infestans* mycelial growth, did not produce melanoid pigments (Supplementary Table S2), suggesting that anti-*Phytophthora* activity was not always associated with this property. Regarding the few isolates not belonging to the *Streptomyces* genus, the inhibition activity of strain B184 (*A. cyanogriseus*) could not be evaluated because of its inability to grow on V8, while the *S. espanaensis* strain (B95) and the *Nocardiopsis flavescens* (B121) strain were almost inactive against *P. infestans* (12% vs. 15% inhibition percentages, respectively). A siderophore production assay was performed in order to evaluate the putative contribution of siderophores in the overall activity against *P. infestans*, as competition for iron is an important determinant of biocontrol activity. Siderophore producers showed an orange halo around their colony on V8-based CAS medium (Supplementary Figure S4). From the entire collection, only nine strains were able to produce siderophores (labeled with an orange asterisk in Figure 1), six of them were isolated from the rhizosphere of *Azadirachta indica* collected from Al Bageir, Gezira State (B132, B134, B140, B144, B145, and B150), two were isolated from the rhizosphere of *Acacia oerfota* collected from Er Roseires, Sennar State (B113 and B174), and one was isolated from the Nile river sediments collected from Khartoum State (B9). The siderophore producers exhibited different inhibition activities on *P. infestans* mycelial growth, ranging from more than 70% inhibition (B145 and B174) to ca. 65% (B113, B144), ca. 25%–30% (B9, B132, B134, B140), and to as low as 14% (B150). This lack of correlation between siderophore production and anti-*Phytophthora* activity suggests the involvement of other bioactive compounds in the inhibition of the oomycete.

### Actinomycetes Are Causing Different *Phytophthora infestans* Morphologies

**Figure 3:**
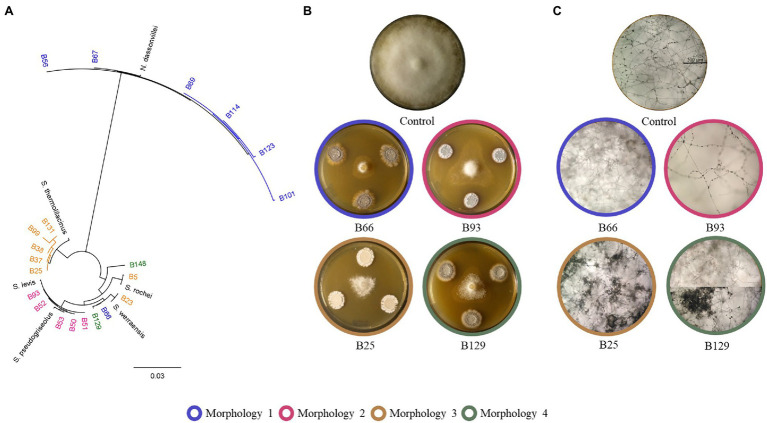
Different morphologies of *Phytophthora infestans* caused by Actinomycetes. **(A)** Phylogenetic tree showing the clusters of the strains, which caused the four described morphologies. **(B)** Representative plates showing the four different morphologies. **(C)** Microscopic pictures showing the altered mycelium and sporangia morphologies caused by the Actinomycetes strains.

Different morphologies of *P. infestans* mycelial growth were observed upon co-inoculation with some of the Actinomycetes isolates, regardless of the extent of growth inhibition. In one of the observed morphologies, *P. infestans* was growing under the agar and no sporangia were observed microscopically (Figure 3, blue circles and Supplementary Figure S5). This morphology was caused by one *Streptomyces* strain (B66), as well as by all the *N. dassonvillei* strains (data not shown). A second morphology was observed upon co-cultivation with five *Streptomyces* strains (B50, B51, B52, B53, B93), which clustered together with *S. pseudogriseolus* (Figure 3A). In the presence of these strains, the growth of *P. infestans* seemed weak compared to the control, with much lesser dense mycelium that still carried sporangia (Figure 3, pink circles and Supplementary Figure S5). Seven *Streptomyces* strains (B5, B23, B25, B37, B38, B99, and B131), five of which clustered together with *S. thermolilacinus*, caused a “frozen” *P. infestans* mycelium morphology, with hyphal clumps and fewer sporangia (Figure 3, brown circles and Supplementary Figure S5). Finally, two *Streptomyces* strains (B129 and B148) induced a mixed morphology between the first (growth under the agar) and the third (“frozen”) in two different zones of the oomycete colony (Figure 3, green circles and Supplementary Figure S5). Those two strains were both isolated from the rhizosphere of *Balanites aegyptiaca* but from two different locations.

### Leaf Disc and *in planta* Assays

**Figure 4:**
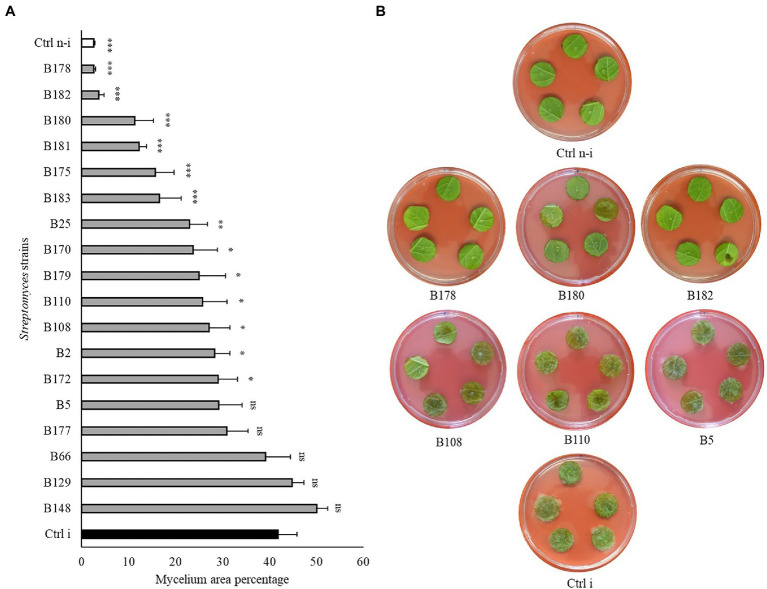
Leaf disc assay of Streptomyces strains against *Phytophthora infestans*. **(A)** Bar chart showing average mycelium growth area percentage (measured by Image J). Bars are averages of 15 leaf discs from two independent experiments with standard errors. Asterisks represent statistically significant differences in comparison to the non-treated, infected control (^***^p < 0.001, ^**^p < 0.01, and ^*^p < 0.05). **(B)** Representative pictures of the leaf discs from the non-treated, infected (Ctrl i) and non-infected controls (Ctrl n-i), as well as from strains showing different levels of disease inhibition. Pictures were taken 5 days after infection.

**Figure 5:**
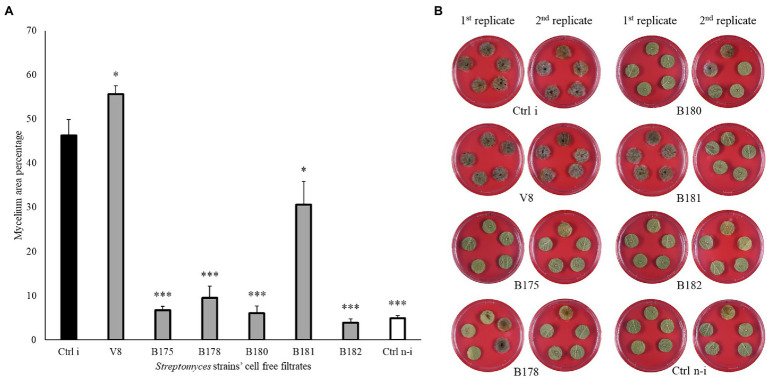
Leaf disc assay of Streptomyces’ cell-free filtrates against *Phytophthora infestans*. **(A)** Bar chart showing average mycelium growth area percentage (measured by Image J). Bars are averages of 20 leaf discs from two independent experiments with standard errors. Asterisks represent statistically significant differences in comparison to the non-treated, infected control (^***^p < 0.001 and ^*^p < 0.05). **(B)** Representative pictures of the leaf discs from the non-treated, infected (Ctrl i) and non-infected controls (Ctrl n-i), as well as from 50% of strains’ cell-free filtrate showing different levels of disease inhibition. Pictures were taken 5 days after infection.

To assess whether the strains showing highest *in vitro* activities against *P. infestans* would also be able to restrict the pathogen development on its host plant, a leaf disc experiment was carried out for the 18 *Streptomyces* strains which showed *in vitro* inhibition potential higher than 90%. A large majority of these isolates were also able to significantly reduce *P. infestans* progression on leaf discs (Figure 4 and Supplementary Figure S6) compared to the non-treated, infected control (Ctrl i). Six strains (B175, B178, B180, B181, B182, and B183) induced full (B178, B182) or highly significant (B175, B180, B181 and B183) protection of the leaf discs against infection by *P. infestans.* It should be noted that these six strains showing strongest leaf disc protection all belonged to the clade of melanoid pigment producers, while four of the five strains active *in vitro* but inactive *in planta* (B5, B66, B129, B148) belonged to different clades. When the cell-free filtrate (CFF) of the five most protective strains was used instead of the spores (Figure 5 and Supplementary Figure S7), it was in almost all cases as efficient as the spores themselves, with the exception of one strain (B181), which completely inhibited the disease in one replicate plate but not on the second one (Figure 5 and Supplementary Figure S7).

**Figure 6:**
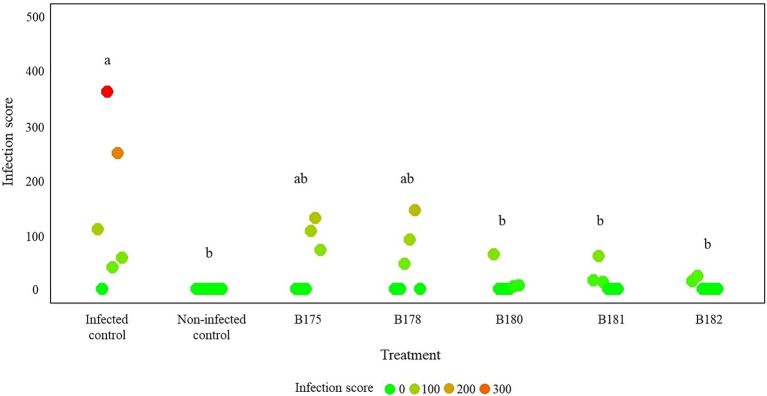
Results of a full plant infection assay on greenhouse-grown potato plants treated with *Streptomyces* spores and infected with *Phytophthora infestans* zoospores. The infection assay was carried out on three plants per treatment in two independent experiments. Infection was assessed 14 days after infection and scores were calculated according to the procedure detailed in section “Materials and Methods” and illustrated in Supplementary Figure S1. Letters indicate significant differences between treatments according to ANOVA followed by Tukey’s honestly significant difference (HSD) tests (*p* < 0.05).

As a last step to come closer to natural conditions, we tested the protective effects of the five best strains by spraying their cell suspensions on fully grown potato plants prior to infecting them. Although the infection rate in the non-treated, infected control strongly varied between the three replicate plants in the two independent experiments, a clear tendency toward higher protection of the treated plants was observed (Figure 6 and Supplementary Figure S8). From the two best inhibitors on leaf discs, B182 performed very well in the two independent experiments, while B178 efficiently protected two plants in the first experiment but only one plant in the second one. Interestingly, the few leaves showing infection in B182-treated plants were young leaves appearing only after the bacterial treatment. It therefore seems likely that direct inhibition of *P. infestans* rather than induced resistance was the cause of protection, which is corroborated by the full leaf disc protection observed upon treatment with B182 spores or CFF (Figures 4, 5).

## Discussion

Soils are one of the most diverse environments when considering microbes (Torsvik and Øvreås, 2002). In the rhizosphere, mutualistic relationships with plants are established, plants providing microbes with organic carbon, while microbes help plants to grow and to tolerate abiotic and biotic stresses. In terms of biotic stress alleviation, microbes can control plant diseases through different mechanisms such as competitive rhizosphere colonization, induction of systemic resistance, or production of antimicrobial metabolites (Reddy, 2014). Since Actinomycetes are known to produce a wide range of specialized metabolites, we anticipated that they could represent interesting biocontrol agents to fight plant diseases. We therefore isolated a new collection of 175 Actinomycete strains from 18 soil samples collected from various locations in Sudan. Taxonomic identification of the strains, based on 16S rDNA sequencing, revealed one *A. cyanogriseus*, one *S. espanaensis*, seven *Nocardiopsis* strains and a vast majority of strains (166) belonging to the genus *Streptomyces*. The dominance of the *Streptomyces* over the other Actinomycetes might be due to their easy isolation on selective media. El-Tarabily and Sivasithamparam (2006) reviewed the Non-Streptomycete Actinomycetes (NSA) and discussed their potential as biocontrol agents and plant growth promoters. They highlighted the need for targeted isolation methods to selectively isolate NSA, as these might not be growing or might be outcompeted on traditionally used media. It was difficult to identify the isolated *Streptomyces* to the species level with their 16S rDNA sequences, as the *Streptomyces* genus contains more than 500 species (Labeda, 2011) which cannot be properly resolved on the sole basis of the relatively conserved 16S rRNA encoding gene. Other identification tools such as MLST (multilocus sequence typing) or polyphasic taxonomy would be needed to identify our isolated *Streptomyces* to the species level (Guo et al., 2008; Yan et al., 2018; van der Aart et al., 2019). Nevertheless, different clades within the *Streptomyces* genus could be identified, which grouped strains that also shared some phenotypic features such as melanoid pigment production or induction of specific phenotypes in mycelial growth of *P. infestans* (Figures 1, 3).

In view of their prolific synthesis of specialized metabolites, Actinomycetes have been repeatedly described to inhibit the growth and development of various plant pathogenic organisms (Abdallah et al., 2013; Chen et al., 2016; Djebaili et al., 2021). Among the many pathogens threatening plant growth and health, the oomycete *P. infestans* is one of the most important ones (Kamoun et al., 2015). As a first step in our attempt to characterize the biological control potential of our newly assembled rhizosphere- and soil-borne Actinomycete collection, we exposed the oomycete to the different isolates in *in vitro* confrontational dual assays. Most isolates significantly reduced the mycelial growth of *P. infestans*, some of them to an extent close to full inhibition (98%). Almost all active strains belonged to the *Streptomyces* genus, while isolates affiliated with the species *N. flavescens* and *S. espanaensis* showed very little activity against *P. infestans* mycelial growth. This contrasts with a recent study reporting anti-*Phytophthora* activity of a *Saccharothrix texasensis* strain isolated from the potato rhizosphere (Feng et al., 2021). Moreover, all strains belonging to the species *N. dassonvillei* also strongly inhibited *P. infestans* mycelial growth, but their opportunistic human pathogen status precludes their use as biocontrol agents and they were therefore not investigated any further in the present study. Regarding the *Streptomyces* strains, we observed that all those able to produce melanoid pigments on V8 showed high anti-*Phytophthora* activity. Further studies are needed to investigate whether the active compound(s) is/are produced by the melanoid pigment pathway or whether the strains’ activity is linked to unrelated molecules commonly produced by this group of phylogenetically related strains.

Beyond the observed growth inhibition, this study reported for the first time the ability of *Streptomyces* spp. to affect the morphology and growth behavior of *P. infestans* hyphae, a property which was not linked to the extent of mycelial growth inhibition but generally encompassed reduced sporangia formation, which might be of relevance for crop protection, as asexually formed sporangia and their released zoospores are the major source of disease spread during late blight epidemics (Leesutthiphonchai et al., 2018). The association of each of the four observed morphologies with groups of phylogenetically closely related strains could help to identify the active compounds involved in inducing such change in *P. infestans* growth behavior, e.g., by comparative metabolomics on the cell-free filtrates of these strains and on that of closely related strains not inducing the specific morphology. Moreover, other experiments with specific setups could help us to assess the contribution of volatile organic compounds to the overall activity of the Actinomycetes we observed in this study. Indeed, in addition to soluble compounds, Actinomycetes are famous for producing a volatile odorous terpene named geosmin, which is responsible for the earthy smell of soil (Schöller et al., 2002), and which has been recently shown to be involved in spore dispersal by bacterivorous insects (Becher et al., 2020). Beyond geosmin, Actinomycetes have been reported to produce complex bouquets of volatiles with antimicrobial properties of relevance for crop protection, especially against post-harvest diseases (Li et al., 2010; Wang et al., 2013; Boukaew et al., 2018; Lyu et al., 2020), and these compounds might therefore also contribute to the inhibition of *P. infestans* mycelial growth observed in our experiments, especially since earlier studies revealed the sensitivity of this oomycete to bacterial volatiles (De Vrieze et al., 2015; Hunziker et al., 2015; Chinchilla et al., 2019; Anand et al., 2020). Independently of the chemical properties (volatile vs. non-volatile) of the metabolites, our anti-*Phytophthora* activity survey of over 160 new *Streptomyces* isolates spanning diverse subclades of this genus and of the specific phenotypes they induced will provide a solid basis for the identification of new antimicrobial compounds of potential relevance for sustainable crop protection. Although members of this clade have been intensively studied for their production of specialized metabolites, comparative genomics studies recently identified a vast reservoir of biosynthetic gene clusters for potentially new natural products (Doroghazi and Metcalf, 2013; van Bergeijk et al., 2020).

In terms of crop protection against diseases, the *in vitro* assays of a pathogen’s mycelium growth, while it provides a valuable high-throughput screening method, is not always correlated to efficacy *in planta* (Guyer et al., 2015; De Vrieze et al., 2018). To assess whether the strong inhibiting activity observed *in vitro* for some strains would also translate into inhibition of plant disease on leaf material, we performed a leaf disc assay with the 18 best strains. Six of them, all belonging to the afore-mentioned cluster of melanoid pigment producers, led to a highly significant reduction of the disease symptoms, two of which even conferring full protection. When the five strains inducing highest disease inhibition on leaf discs were tested in full plant assays, their protective potential was confirmed, although the generally low infection even in control plants does not warrant similar protection under field conditions where disease pressure can be very high. Among the strains which were highly efficient in reducing *P. infestans* mycelial growth *in vitro,* many (including the best inhibitors B5 and B108) did not trigger reduction of disease symptoms when inoculated on leaf discs. This could be due to their antagonistic activity being specifically targeting mycelial growth but not other development stages, such as zoospore germination or *in planta* infection. In an earlier study focusing on potato-associated *Pseudomonas,* we also observed a discrepancy between the ability to inhibit mycelial growth *in vitro* and disease spread on leaf discs (De Vrieze et al., 2020). In addition, as our strains were isolated from soil and rhizosphere samples, this lack of efficacy on leaf discs could suggest their inability to grow properly in the phyllosphere environment which offers very different growth conditions for microbes (Vorholt, 2012). In such cases, one solution to still harness the anti-oomycete potential of the strains would be to use their metabolites. When we compared the disease-inhibiting efficacy of the spore suspensions with that of the respective cell-free filtrates, we found similar reduction of disease spread on leaf discs (Figures 4, 5). This suggests that combining leaf applications of Actinomycete culture extracts with soil drenching of the biocontrol agents to lower tuber infection (Feng et al., 2022) might be a valid strategy to efficiently control diseases such as potato late blight infecting both above- and below-ground plant parts.

In summary, our study not only confirmed the extended *in vitro* inhibitory potential of Actinomycetes on a disease-causing agent of highest economical relevance such as *P. infestans*, but also highlighted the protective potential of these strains and their culture extracts in leaf disc experiments, with a first and promising assessment of their performance in whole plant infection assays. More specifically, we identified a subclade in the *Streptomyces* genus (phylogenetically related to the species *S. globosus, S. achromogenes* and *S. antibioticus*) enriched in strains which produced melanoid pigments and were particularly efficient inhibitors of *P. infestans* in both *in vitro* and leaf disc assays. Beyond this specific clade, other subclades in the genus contained strains which led to distinctive inhibitory morphologies indicative of impaired hyphae and sporangia formation of putative relevance for disease control. The occurrence in our newly assembled strain collection of isolates genetically closely related but not causing these morphologies will favor the identification of the responsible active compounds by future comparative metabolomic analyses. Although the observed *P. infestans* inhibition rates by some of our newly described *Streptomyces* strains are impressive (98% of *in vitro* inhibition and full disease prevention in leaf discs and full plants), the road to an efficient biocontrol solution in the field is still very long. The next step on the path toward implementing these strains into crop protective measures would be to test their efficacy at a larger scale in greenhouse conditions as well as in field trials involving different potato cultivars and modes of application (spores vs. cell-free filtrates, soil drench vs. foliar application). Moreover, combination of different strains rather than the use of single isolates has been proposed as a promising strategy in earlier studies and was recently shown to be the only efficient solution for tuber protection under field conditions (Feng et al., 2022). Finally, elucidating the chemical nature of at least some of the compounds underlying the observed anti-*Phytophthora* activity both in terms of *in vitro* mycelial growth and of *in planta* disease spread inhibition will be the focus of our future studies and could potentially also lead to new bioderived molecules of interest for sustainable crop protection.

## Data Availability Statement

The original contributions presented in the study are included in the article/Supplementary Material, further inquiries can be directed to the corresponding author.

## Author Contributions

OA, SY, ME, AE, and LW designed the research. OA performed all experiments with help from FL’H, FG, and MV. OA and LW wrote the paper with help from all authors. All authors contributed to the article and approved the submitted version.

## Funding

This work was supported by the Swiss National Science Foundation (grant 179310 to LW) and the Ministry of Higher Education and Scientific Research in Sudan.

## Conflict of Interest

The authors declare that the research was conducted in the absence of any commercial or financial relationships that could be construed as a potential conflict of interest.

## Publisher’s Note

All claims expressed in this article are solely those of the authors and do not necessarily represent those of their affiliated organizations, or those of the publisher, the editors and the reviewers. Any product that may be evaluated in this article, or claim that may be made by its manufacturer, is not guaranteed or endorsed by the publisher.
