# Supplementary material for: Evaluating the Antagonistic Potential of Actinomycete Strains Isolated From Sudan’s Soils Against Phytophthora infestans
Source: Front Microbiol. 2022 Jun 29;13:827824. doi: 10.3389/fmicb.2022.827824 (PMC9277107; doi:10.3389/fmicb.2022.827824)
Supplement: Supplementary file 1 [file Data_Sheet_1.pdf]

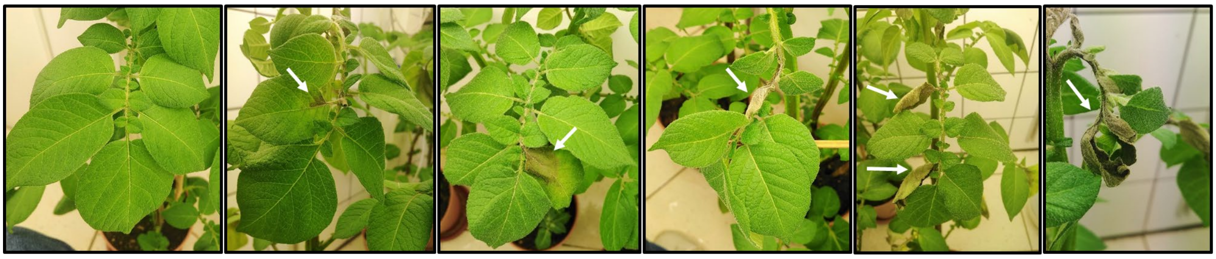

0 1 2 3 4 5

Figure S1: Representative pictures of the scoring scale used to assess disease progress of greenhouse potato plants infected with *Phytophthora infestans* zoospores. Score 0 stands for no infection, score 1 for an infected leaf with a lesion diameter smaller than one cm, score 2 for a lesion diameter larger than one cm, score 3 for a dead leaflet, score 4 for more than one dead leaflet and score 5 for an entirely dead leaf.

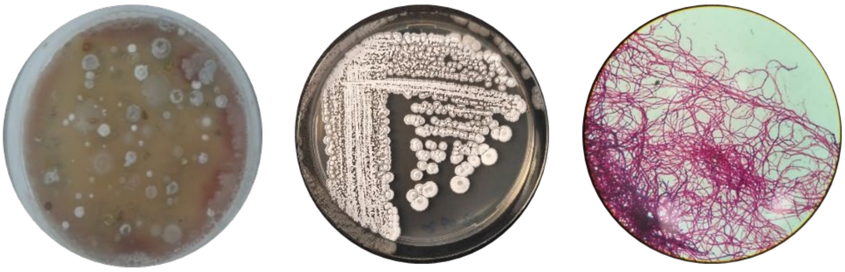

A B C

Figure S2: (A), Representative picture of the isolated Actinomycetes on SCNA medium. (B), Purified strain on SCNA. (C), Representative microscopic picture of Gram-positive filamentous mycelium of isolated Actinomycetes.

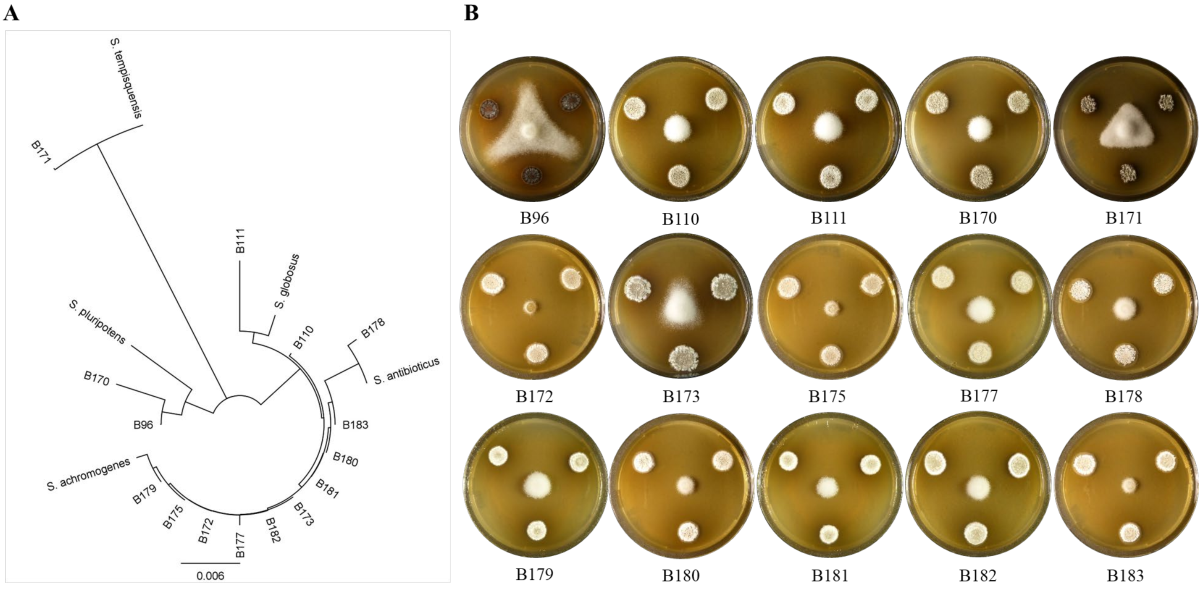

Figure S3: (A), Phylogenetic tree of the 15 melanoid pigment producers on V8 medium. The tree was constructed using Geneious software and based on ca. 1050 bp of the 16S rDNA gene sequence. Reference sequences from different species of the *Streptomyces* (*S.*) genus corresponding to the following accession numbers were included: *S. achromogenes* (LC535408.1), *S. antibioticus* (EU841627.1), *S. globosus* (HM230830.1), *S. pluripotens* (KX129895.1), and *S. tempisquensis*. (B), Plates of the 15 strains showing various *P. infestans* growth percentages compared to the control (100% growth percentage).

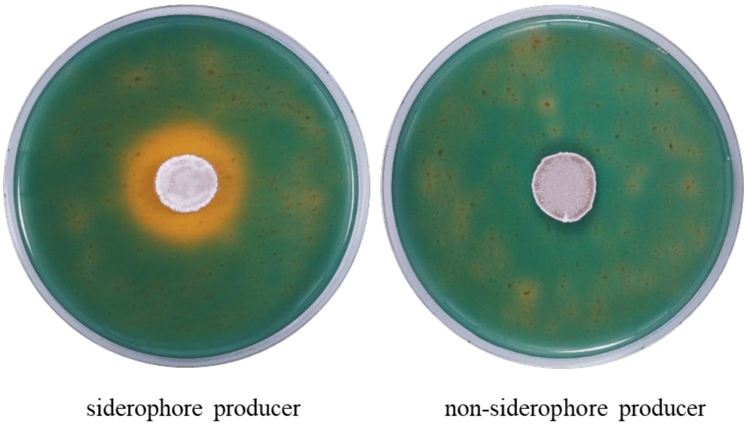

Figure S4: Representative pictures of siderophore and non- siderophore producer Actinomycete strain on V8 medium, the orange halo indicates siderophore production.

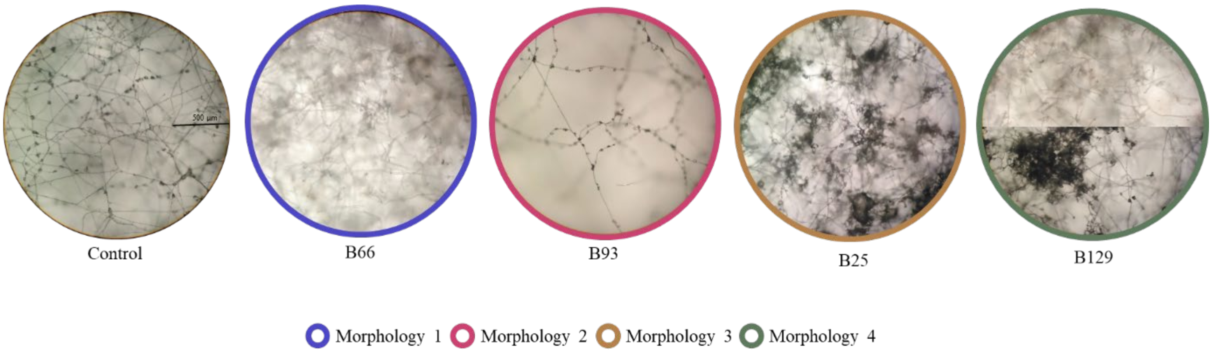

Figure S5: Microscopic pictures showing the altered mycelium and sporangia morphologies of *Phytophthora infestans* when co-inoculated with Actinomycetes strains.

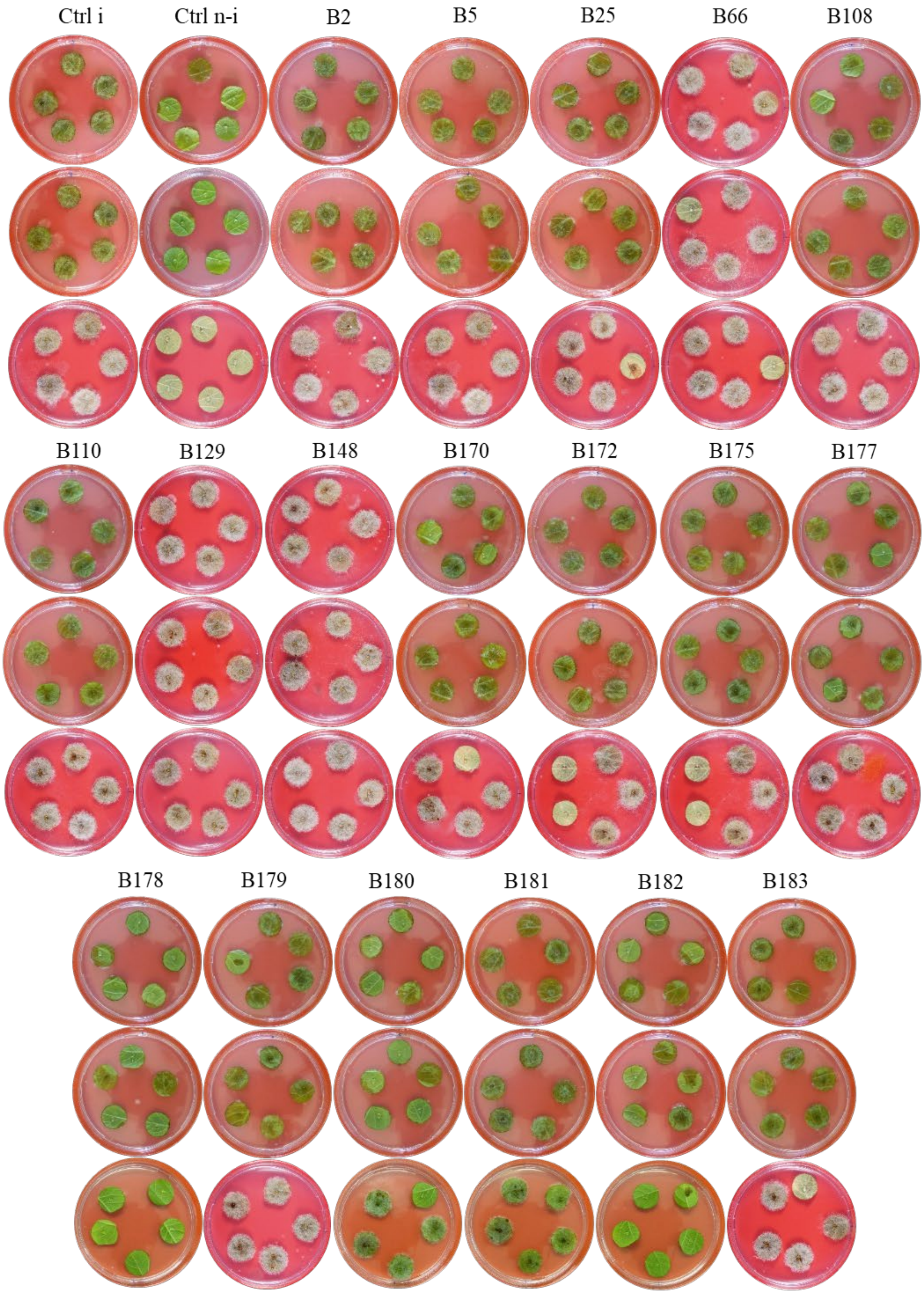

Figure S6: Pictures of leaf disc assay of *Streptomyces* strains against *Phytophthora infestans* showing different levels of disease inhibition. Pictures were taken five days after infection.

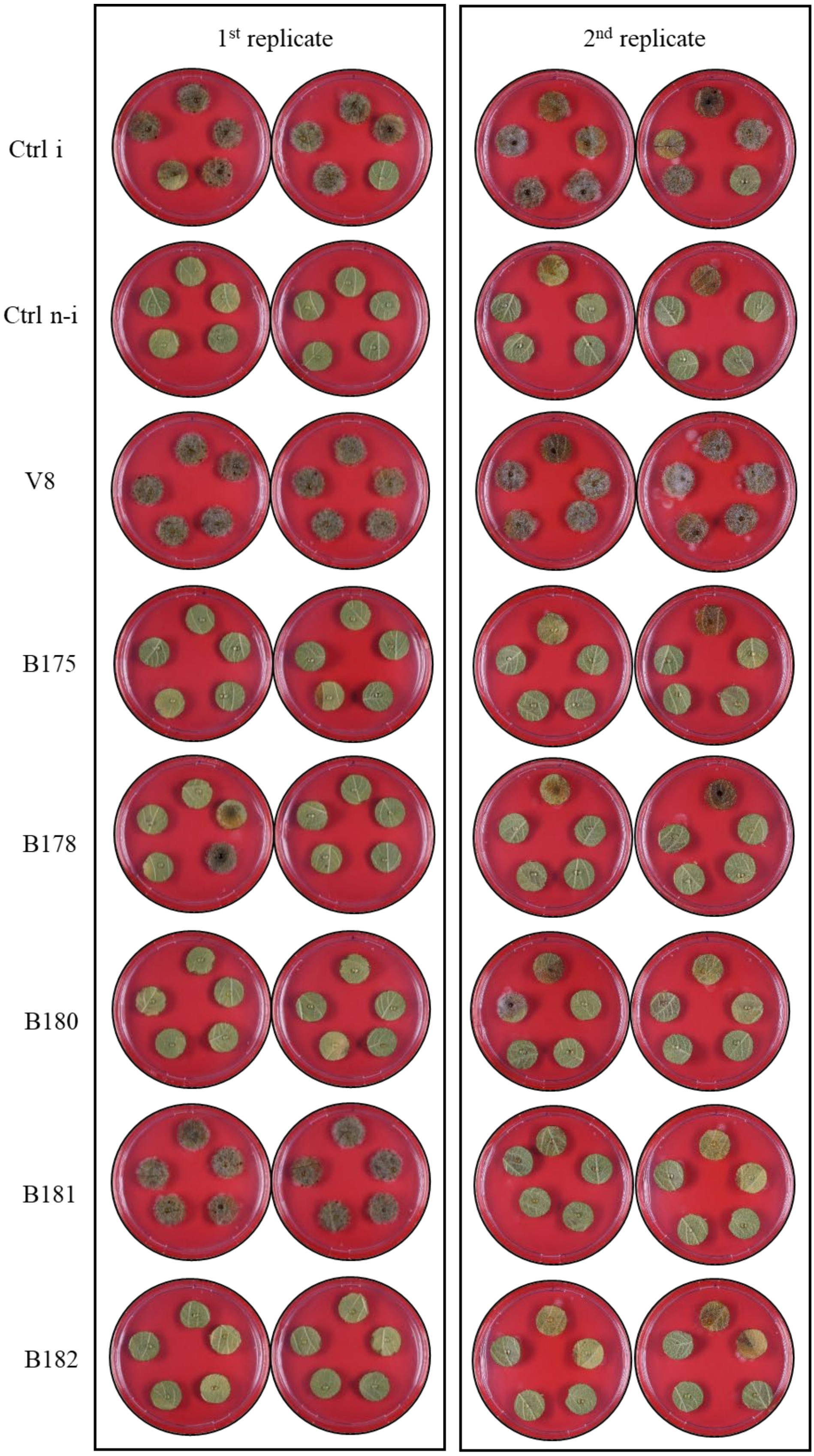

Figure S7: Pictures of leaf disc assay of *Streptomyces'* cell-free filtrates (50%) against *Phytophthora infestans* showing different levels of disease inhibition. Pictures were taken five days after infection.

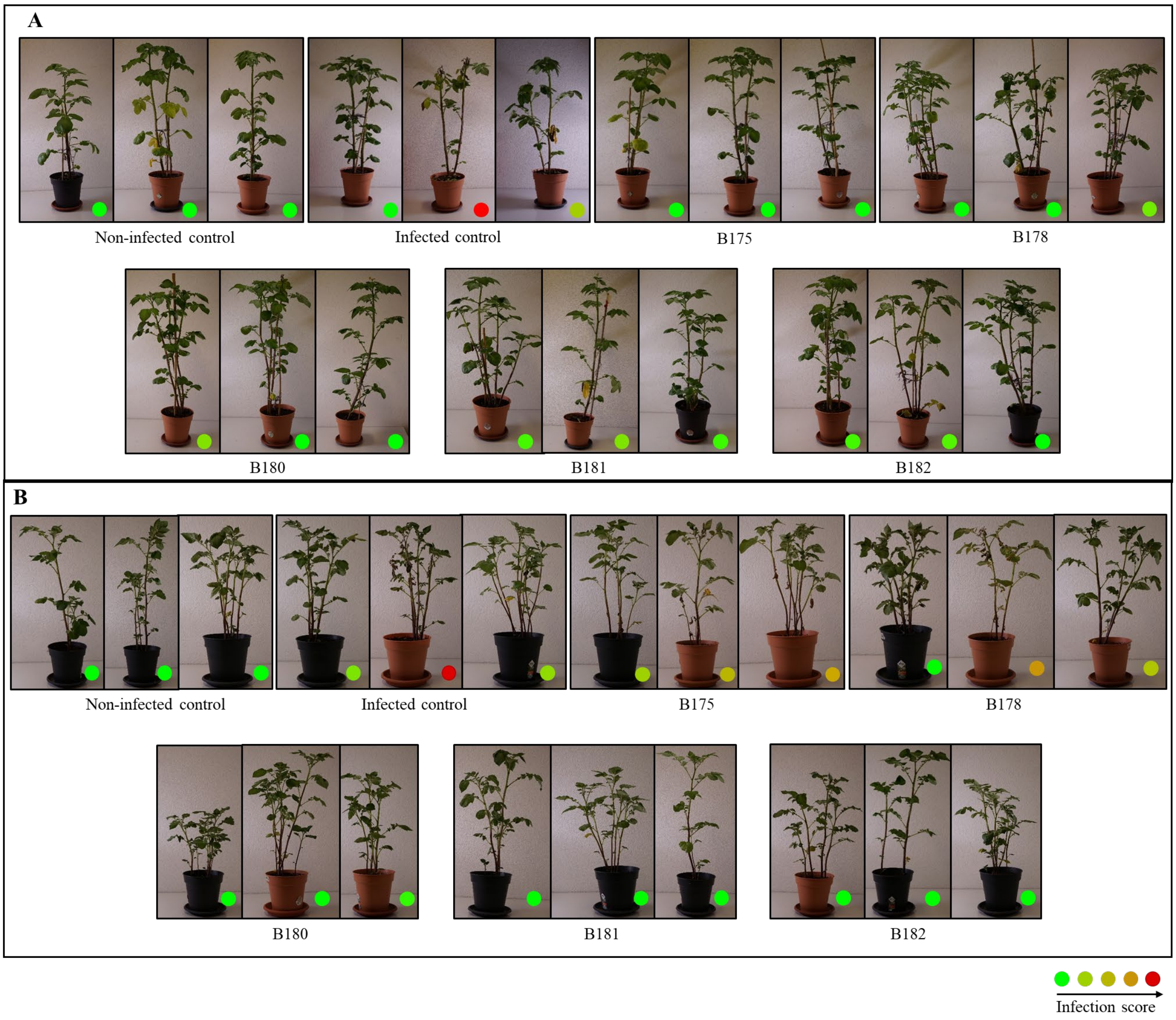

Figure S8: Pictures of a full plant infection assay on greenhouse-grown potato plants treated with *Streptomyces* spores and infected with *P. infestans* zoospores. The infection assay was carried out on three plants per treatment in two independent experiments (A and B). Infection was assessed 14 days after infection and scores were calculated according to the procedure detailed in Material and Methods.
